# Cyanoacrylate glue embolisation for varicose veins – A novel complication

**DOI:** 10.1177/0268355520901662

**Published:** 2020-01-28

**Authors:** Benjamin J Langridge, Sarah Onida, Justin Weir, Hayley Moore, Tristan RA Lane, Alun H Davies

**Affiliations:** 1 Academic Section of Vascular Surgery, Department of Surgery and Cancer, Imperial College London, UK; 2 Imperial Vascular Unit, Imperial College Healthcare NHS Trust, London, UK; 3 Department of Pathology, Imperial College Healthcare NHS Trust, London, UK

**Keywords:** Varicose veins, venous disease, cyanoacrylate glue, complication, safety

## Abstract

**Background:**

Non-thermal non-tumescent methods for varicose vein treatment have rapidly gained popularity in recent years due to clinical efficacy comparable to other endovenous methods, but with a superior safety and tolerability profile. Cyanoacrylate is an adhesive that rapidly polymerises during endovenous treatment to cause rapid occlusion of veins and initiate vein fibrosis.

**Method:**

Cyanoacrylate glue treatment is known to cause complications such as phlebitis, cellulitis and deep vein thrombosis in rare instances. We present the first reported case of cyanoacrylate extravasation with chronic foreign body reaction in a patient nine months after initial treatment.

**Results:**

We discuss the aetiology of this complication, its treatment, patient outcome and its significance to both clinicians and patients.

**Conclusion:**

Cyanoacrylate glue embolisation can, in rare instances, lead to extravasation and chronic foreign body reaction, necessitating surgical intervention. The relative novelty of cyanoacrylate glue embolisation in the treatment of varicose veins requires clinicians to monitor for rare complications during its use in clinical practice. Patients should be aware of the rare risk of glue extravasation and foreign body reaction for fully informed consent prior to treatment.

## Introduction

Cyanoacrylate glue embolisation is a comparatively novel endovenous method for treating varicose veins. Current evidence suggests similar efficacy to other endovenous methods but with a superior safety and tolerability profile.^1^ We report the first documented case of delayed extravasation and chronic foreign body reaction following the use of cyanoacrylate glue and discuss its importance for both clinicians and patients.

## Case

A 54-year-old woman presented with a three-week history of a painless lump in the anteromedial aspect of her left thigh. Nine months previously she had undergone endovenous glue embolisation (VenaSeal Closure System – MedTronic, USA) of her left great saphenous vein (GSV) under local anaesthetic for CEAP C4a chronic venous disease. The patient’s only medical history was previous venous avulsions at a different anatomical location; she used no medications, had a body mass index of 28, and had no history of allergy or hypersensitivity type reactions. During the procedure the proximal GSV was treated twice, with a total of 2.5 ml of glue being used to treat 43 cm of GSV. No anatomical abnormalities were observed and no difficulties were encountered intraoperatively. There had been no immediate complications and the deep venous system was confirmed as patent after treatment was completed. Post-operatively the patient received compression therapy and a single dose of prophylactic low-molecular-weight heparin, as per standard protocol at our institution.

**Figure 1.**
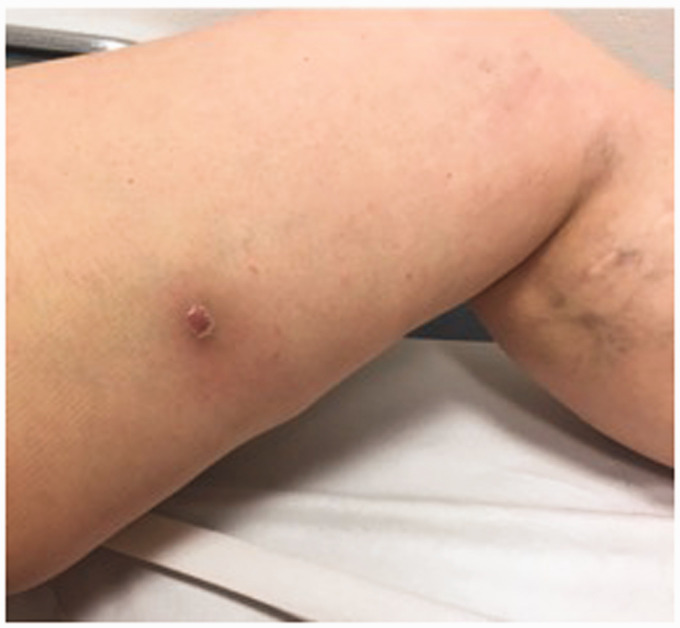
Pre-operative image demonstrating the patient’s erythematous lesion of the mid-thigh.

**Figure 2.**
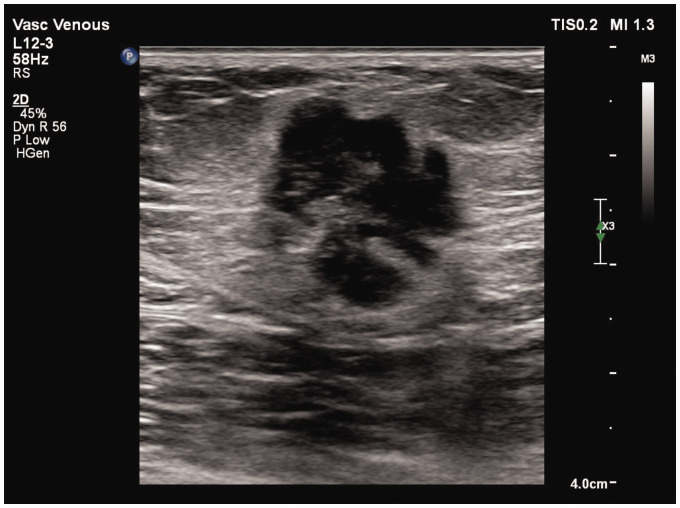
Ultrasound image demonstrating a subcutaneous heterogeneous hypoechoic collection, underlying the erythematous lesion of the patient’s thigh.

Examination of the patient in clinic identified multiple hard, non-tender lumps in the anteromedial aspect of the superior to middle thigh, with one visible area of erythema that had developed progressively over three weeks but no associated discharge (Figure 1). This site was more than 15 cm proximal to the cannulation site. Ultrasound examination of the area confirmed one extravascular, heterogeneous, non-compressible hypoechoic mass associated with the erythematous nodule, with multiple similar intravascular nodules located linearly along the ablated intra-fascial GSV (Figure 2). There were no venous tributaries associated with the masses.

**Figure 3.**
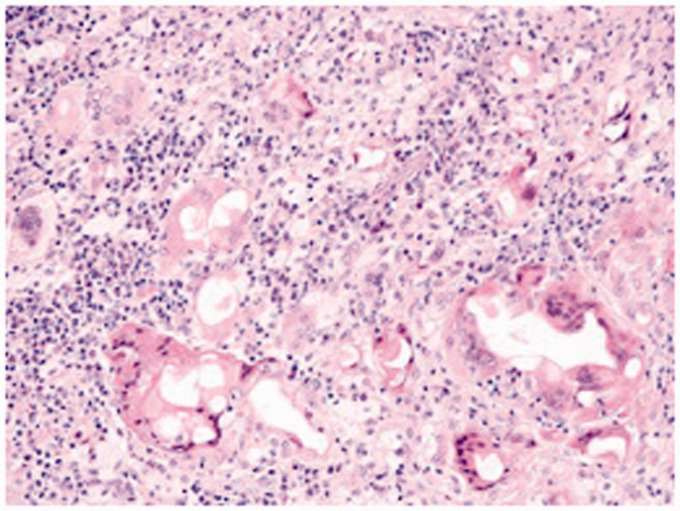
Histology image from the excised tissue (haemotoxylin and eosin; 200× magnification). Multinucleated giant cells can be seen surrounding clear, non-refractile glue, which are in turn surrounded by plasma cells and lymphocytes.

Surgical exploration of the erythematous area was performed under sterile conditions and local anaesthetic in an operating theatre. An elliptical incision was made around the lesion, with a single heterogenous mass of approximately 2 cm × 1 cm being excised in to from the subcutaneous tissue and sent for histological and bacteriological analysis (Figure 3). The excised mass was extravascular, approximately 5 cm from the remaining intravascular nodules within the remnant GSV, and separated from the GSV by a plane of scar tissue. The wound was closed primarily with interrupted 3/0 prolene sutures, and a small drain placed in the cavity. Histological analysis identified florid inflammation of the dermis and subcutaneous tissue, with lymphoid aggregates, eosinophils and foam histiocytes surrounding the extravasated glue, consistent with a foreign body type reaction. No evidence of bacteriological growth nor of allergic reaction was found on testing.

Post-operatively the patient recovered well; at one-month follow-up, the patient was clinically stable with no progression of the remaining lumps, which remained palpable, and the GSV remained occluded. She will continue to be monitored to assess the need for further intervention.

## Discussion

The treatment of varicose veins has changed substantially in recent years, with minimally invasive techniques under local anaesthesia becoming preferred over traditional high ligation and stripping.^2^ Minimally invasive techniques such as radiofrequency ablation have been recommended by the National Institute of Clinical Excellence^3^; however, the use of thermal energy and tumescent infiltration can cause pain, skin burns and skin pigmentation, amongst other problems. Cyanoacrylate glue embolisation is a comparatively novel non-thermal non-tumescent method that is reported to avoid many of these concerns and thus is growing in popularity.

Cyanoacrylate is a liquid adhesive agent that rapidly polymerises when in contact with solutions containing anions such as blood, causing vessel occlusion, inflammation and fibrosis.^4^ Multiple studies have demonstrated that it is highly effective in the treatment of varicose veins, with complete occlusion in over 90% of patients at 12-month follow-up,^5^ suggesting a clinical effectiveness at least equivalent to that of endothermal ablation.^1^

Cyanoacrylate glue also appears to be a safe method of treatment, with a low rate of complications reported in clinical studies.^6^ Reported complications are typically mild and managed either conservatively or on an outpatient basis and include pain, thrombophlebitis, cellulitis, skin hyperpigmentation and deep vein thrombosis.^7,8^ However, clinical studies currently published do not have large patient cohorts, leading to the reasonable concern that they are underpowered to detect rare complications.^6^ Clinicians must therefore be alert to the potential of encountering such rare complications in their practice.

Our patient is the first documented case in the literature of cyanoacrylate glue extravasation and foreign body reaction. The precise mechanism by which the cyanoacrylate glue underwent delayed extravasation is unclear; the original procedure was completed by an experienced clinician in the standard manner with no immediate complications or difficulties. We would suggest a potential mechanism may be a chronic immunological reaction to cyanoacrylate with subsequent damage to the vessel wall; this is supported by emerging clinical and preclinical reports of immunological reactions to cyanoacrylate treatment.^9–11^ An alternative mechanism is that during compression after glue installation, as per the manufacturer’s instructions for use, glue may be compressed into a very small tributary leading to its rupture, extravasation and subsequent foreign body reaction. We would therefore suggest that such compression should be light and applied with great care. This is an important complication particularly due to the involvement of adjacent skin in the inflammatory process, the need for surgical intervention, its spontaneous appearance many months after treatment, along with its negative effect on both cosmesis and patient satisfaction. A clear warning of the possibility of this complication should be given to patients, with clinical review if indicative symptoms should develop.

## Conclusions

Cyanoacrylate embolisation has great potential as a treatment for varicose veins given its clinical efficacy comparable to other endovenous methods, but with an apparent improved safety and tolerability profile. Given the relative novelty of its endovenous use, rare complications are likely to be identified in clinical practice and clinicians must be alert to this risk. Clinicians should consider the rare risk extravasation and foreign body reaction in their decision making, and patients should be informed of this risk prior to treatment.

## Key messages

1. Cyanoacrylate glue embolisation can, in rare instances, lead to extravasation and chronic foreign body reaction, necessitating surgical intervention.
2. The relative novelty of cyanoacrylate glue embolisation in the treatment of varicose veins requires clinicians to monitor for rare complications during its use in clinical practice.
3. Patients should be aware of the rare risk of glue extravasation and foreign body reaction for fully informed consent prior to treatment.
